# Stroke Volume Variation-Guided Goal-Directed Fluid Therapy Did Not Significantly Reduce the Incidence of Early Postoperative Complications in Elderly Patients Undergoing Minimally Invasive Esophagectomy: A Randomized Controlled Trial

**DOI:** 10.3389/fsurg.2021.794272

**Published:** 2021-12-06

**Authors:** Wei Tang, Yuwei Qiu, Huijie Lu, Meiying Xu, Jingxiang Wu

**Affiliations:** ^1^Department of Anesthesiology, Shanghai Chest Hospital, Shanghai Jiao Tong University, Shanghai, China; ^2^Outcomes Research Consortium, Cleveland, OH, United States

**Keywords:** goal-directed therapy, stroke volume variation, minimally invasive esophagectomy, elderly patient, outcome

## Abstract

**Study Objective:** This study aimed to investigate whether stroke volume variation (SVV)-guided goal-directed therapy (GDT) can improve postoperative outcomes in elderly patients undergoing minimally invasive esophagectomy (MIE) compared with conventional care.

**Design:** A prospective, randomized, controlled study.

**Setting:** A single tertiary care center with a study period from November 2017 to December 2018.

**Patients:** Patients over 65 years old who were scheduled for elective MIE.

**Interventions:** The GDT protocol included a baseline fluid supplement of 7 ml/kg/h Ringer's lactate solution and SVV optimization using colloid boluses assessed by pulse-contour analysis (PiCCO™). When SVV exceeded 11%, colloid was infused at a rate of 50 ml per minute; if SVV returned below 9% for at least 2 minutes, then colloid was stopped.

**Measurements:** The primary outcome was the incidence of postoperative complications before discharge, as assessed using a predefined list, including postoperative anastomotic leakage, postoperative hoarseness, postoperative pulmonary complications, chylothorax, myocardial injury, and all-cause mortality.

**Main Results:** Sixty-five patients were included in the analysis. The incidence of postoperative complications between groups was similar (GDT 36.4% vs. control 37.5%, *P* = 0.92). The total fluid volume was not significantly different between the two groups (2,192 ± 469 vs. 2,201 ± 337 ml, *P* = 0.92). Compared with those in the control group (*n* = 32), patients in the GDT group (*n* = 33) received more colloids intraoperatively (874 ± 369 vs. 270 ± 67 ml, *P* <0.05) and less crystalloid fluid (1,318 ± 386 vs. 1,937 ± 334 ml, *P* <0.05).

**Conclusion:** The colloid-based SVV optimization during GDT did not significantly reduce the incidence of early postoperative complications after minimally invasive esophagectomy in elderly patients.

**Clinical Trial Number and Registry URL:** ChiCTR-INR-17013352; http://www.chictr.org.cn/showproj.aspx?proj=22883

## Introduction

The incidence of postoperative complications and mortality related to esophagectomy is relatively high, especially in elderly patients with cardiovascular or lung disease (1). The morbidity rate and 30-day mortality rate were reported to be up to 64% and 5%, respectively (2, 3). Despite the use of minimally invasive techniques and well-controlled anesthesia, the incidence of postoperative complications remains high, reaching approximately one thirds (4, 5). Postoperative complications increase health care costs and extend the length of hospital stay.

The relationship between intraoperative fluid management and postoperative morbidity has been studied for several years in esophageal cancer surgery (6). A U-shaped association was observed between the volume of fluid administered intraoperatively and 30-day mortality, costs, and postoperative length of stay (7). Excessive fluid administration may cause pulmonary congestion, decrease tissue oxygenation and lead to interstitial edema, which may impair anastomotic healing. Meanwhile, hypovolemia can be detrimental for the newly constructed gastric conduit and anastomosis. Until recently, the optimal volume for esophagectomy has not been determined (8, 9).

Goal-directed fluid therapy (GDT) seeks to optimize intraoperative fluid load and improve tissue oxygen delivery. Given that MIE (minimally invasive esophagectomy) has become increasingly widespread, further trials evaluating the efficacy of GDT in patients undergoing MIE are warranted. Some studies have reported that SVV-guided GDT may reduce postoperative complications in open esophagectomy; however, its accuracy has been questioned because of the influence of carbon dioxide pneumothorax and pneumoperitoneum in endoscopic surgery. Whether SVV-guided GDT can improve the prognosis of patients undergoing MIE has not been reported.

Administering a fluid challenge is commonly used during GDT to optimize predefined hemodynamic targets such as SVV. During GDT, the key components of fluid challenge include type of fluid (colloids, often 6% HES), volume (250 mL in GDT studies) and time (10). Whether the colloid-based SVV optimization during GDT reduces postoperative complications should also be addressed.

Herein, we aimed to investigate the efficacy of SVV-guided GDT on postoperative outcomes in elderly patients undergoing MIE compared with conventional fluid management. Specifically, we tested the primary hypothesis that SVV-guided GDT can improve postoperative outcomes.

## Materials and Methods

This trial was a single-center, prospective, randomized, controlled study, with approval from the Shanghai Chest Hospital Institutional Review Board (IRB # KS1622). This trial was registered before subject enrollment began at the Chinese Clinical Trial Registry (ChiCTR-INR-17013352, date of registration, Nov 12, 2017). Written informed consent was obtained from each patient before anesthesia.

Eligible patients were 65 years or older, had American Society of Anesthesiologists (ASA) physical status I to III, had newly diagnosed esophageal carcinoma, and were scheduled for MIE.

Patients were excluded if they had severe cardiovascular diseases (defined as persistent atrial fibrillation, New York Heart Association class III or IV, or acute coronary syndrome, or hemodynamic instability or persistent ventricular tachyarrhythmia), were allergic to anesthetics, had end-stage renal diseases requiring dialysis, were contraindicative to femoral artery cannulation, or body mass index > 30 kg/m^2^. Patients were also excluded if they had severe organ infection (including but not limited to septicemia), had coagulopathy, or used immunosuppressive drug therapy before surgery.

### Randomization and Masking

Patients were randomized 1:1 into two groups using a set of computer-generated random numbers kept in sealed envelopes by an investigator not involved in clinical care. Envelopes were opened shortly before anesthetic induction. One investigator was unmasked of the allocated intervention and obtained intraoperative data; the other remained unaware of the interventions and assessed postoperative outcomes. The random allocation was also masked from patients, surgeons, and the ward physicians.

### Intervention Protocol

Patients were randomly allocated 1:1 into two groups: the GDT group and the conventional group. In the GDT group, a pulse-contour analysis catheter (PiCCO^TM^, pv2014L16-A, 4F) was inserted into the femoral artery. Hemodynamic parameters, including SVV, cardiac index (CI), end diastolic volume index (EDVI) and stroke volume index (SVI), were continuously monitored by PULSION (PULSION Medical Systems SE, PC-4000). The extravascular lung water index (EVLWI) was measured by the thermal dilution method (right internal jugular vein injection of 10 ml cold 0.9% saline).

In the GDT arm, the strategy consisted mainly of SVV optimization. Patients were given baseline infusion with 7 ml/kg/h [actual body weight (ABW)] crystalloids (Ringer's lactate solution). Colloid boluses (6% HES 130/0.4, Voluven, Fresenius Kabi, Germany) were used intraoperatively to optimize SVV: when SVV exceeded 11%, colloid was infused at a rate of 50 ml per minute; if SVV returned below 9% for at least 2 minutes, then colloid was stopped. Hydroxyethyl starch was used as the primary colloid solution.

In the control arm, patients received conventional fluid management without pulse-contour analysis (PiCCO^TM^). Fluid was administrated as follows: the total amount of fluid requirements = compensatory intravascular capacity expansion + physiological demand + cumulative loss + continuous loss + third space loss. Compensatory intravascular volume expansion was supplemented with 5 ml/kg Ringer's lactate solution. Physiological requirements and cumulative loss were supplemented with Ringer's lactate solution according to the “4-2-1 formula.” Volumes are calculated using a weight-based infusion rate: for the first 10 kg, 4 ml/kg/h, for the next 10 kg, 2 ml/kg/h and 1 ml/kg/h for each kilogram thereafter (11–13). Continuous loss was given with colloid (6% HES 130/0.4, Voluven, Fresenius Kabi, Germany) according to intraoperative blood loss, and third space loss was replenished with 5 ml/kg Ringer's lactate solution.

During the operation, the amount of blood loss and transfusion was recorded. If bleeding exceeded 30% of the patient's blood volume, blood loss was compensated with fresh frozen plasma, red blood cells and hydroxyethyl starch. Norepinephrine (0.1–0.5 μg/kg/min) was given when intraoperative mean arterial pressure was lower than 60 mmHg after bleeding and cardiac compression were excluded.

### Anesthesia Protocol

None of the patients received premedication. Patients were monitored with electrocardiography (ECG), invasive blood pressure, pulse oximetry, ETCO_2_ and bispectral index (BIS). Invasive blood pressure monitoring was achieved by radial artery cannulation and right internal jugular venous catheterization.

General anesthesia was induced with 0.6 μg/kg sufentanil and a target-controlled infusion of 4 μg/ml propofol. Rocuronium 0.6 mg/kg was given to facilitate single-lumen tube intubation. Propofol administration was adjusted to target a bispectral Index of 40–60. Sufentanil 0.2 μg/kg was added intravenously to provide sufficient intraoperative analgesia.

During mechanical ventilation, the fraction of inhalation oxygen concentration (FiO_2_) was set to 100%, and tidal volume (Vt) was adjusted according to predicted body weight (PBW): PBW for males = 50 kg + 2.3 kg × (Height [in] – 60); PBW for females = 45.5 kg + 2.3 kg × (Height [in] – 60). Volume control mode was used with a Vt of 6.0 ml/kg PBW and a respiratory rate at 12/min with I: E 1:1.5. FiO_2_ was adjusted to 60% when patients were changed to supine position to receive abdominal procedure. Then, Vt was set to 8.0 ml/kg PBW, with RR set to 12/min and I: E at 1:1.5. Arterial carbon dioxide (PaCO_2_) was maintained at 35–45 mmHg, and SpO_2_ was above 95%. The cerebral oxygen saturation of the forehead was monitored by a near infrared cerebral oxygen saturation monitor (MNIR-P100 Mingxi, Chongqing). A forced air warming blanket was used to achieve intraoperative nasopharyngeal temperatures above 36.5°C. In all patients, the composite of sufentanil 1.5 μg/kg was diluted with saline into 100 ml in a patient-controlled intravenous analgesia pump and infused at a rate of 2 ml/h, a demand dose of 0.5 ml bolus and a locking time of 15 minutes.

### Surgical Protocol

All patients received McKeown MIE surgery (three-stage esophagectomy through the modified McKeown thoracoscopic, laparoscopic and cervical approach). Patients were positioned in the left lateral decubitus position for thoracic procedures. A standard right video-assisted thoracoscopic surgery (VATS) with CO_2_ insufflation at 8 mmHg was implemented. After thoracic procedures were completed, patients were changed to the supine position and placed in reverse Trendelenburg position for an abdominal approach. A standard laparoscopic approach with CO_2_ insufflation at 14 mmHg was implemented. The esophagus was anastomosed by the left neck.

### Measurements

Demographic and intraoperative variables were collected. The parameters of SVV, CI, SVI, and EVLWI, arterial blood gas and the cerebral oxygen saturation were collected at 5 time points: after anesthesia induction, immediately after the initiation of artificial pneumothorax, 30 min after artificial pneumothorax establishment, 30 min after artificial pneumoperitoneum establishment and at the end of the surgery. The duration of anesthesia, intraoperative urine output, blood loss, total intraoperative fluid administration, intraoperative crystalloid and colloidal volume, and the number of patients who used norepinephrine were recorded. Cardiac troponin I (cTnI) was monitored before the surgery, on the 1st and 3rd days after surgery.

### Outcomes

The primary outcome was the incidence of postoperative complications before discharge. All complications were identified from the patient's electronic medical record and clinical follow-up. We defined postoperative complications using a predefined list, including pulmonary complications, anastomotic leakage, hoarseness, chylothorax, myocardial injury and all-cause in-hospital mortality. Postoperative pulmonary complications were defined according to the Melbourne Group Scale (14) (Supplementary Table 1). Postoperative myocardial injury was defined as cTnI ≥ 0.06 μg/ml (99% UPL) on the 1st or 3rd day after surgery.

Secondary outcomes included intraoperative hemodynamic parameters, cerebral oxygen saturation, blood gas, total volume of fluid administration, volumes of crystalloid and colloid, intraoperative blood loss, duration of surgery, urine output on the surgical day and the 1st day after surgery, and length of postoperative hospital stay.

### Statistical Analysis

Data are presented as the means ± SD, Median [IQR] and number (%). We used T-test for two normally distributed continuous outcomes, Mann-Whitney U test for nonparametric outcomes, and Chi-square tests or Fisher's exact test to compare the incidence of postoperative complications and categorical variables, Median [IQR]. Unadjusted and adjusted regression analyses were conducted to estimate the relative risk (RR) with 95% confidence intervals (CIs) for the incidence of postoperative complications. We used repeated measures ANOVA to test continuous variables at different time points.

Our minimal sample size was determined on the basis of a previous study after esophageal surgery in our center (15). The predicted incidence of a composite of postoperative complications was 40% in conventional treatment. Among postoperative complications, pulmonary complications, anastomotic leakage and myocardial injury constituted mostly which may benefit from optimized fluid management. This was a preliminary study therefore we set the expected hazard ratio of 0.25 (75% relative reduction) in the composite of postoperative complications. We assumed that SVV-GDT management can lead to the incidence of postoperative complications from 40% to 10%. The sample size was estimated to be 60 (30 per group) by the G^*^Power 3.1.9.2 software using a power of 80% and alpha of 0.05. To allow for a 15% drop-out rate, we planned to enroll 75 patients without interim analyses.

Statistical analyses were performed using SPSS version 20 (IBM, Chicago, IL, USA).

## Results

**Figure 1:**
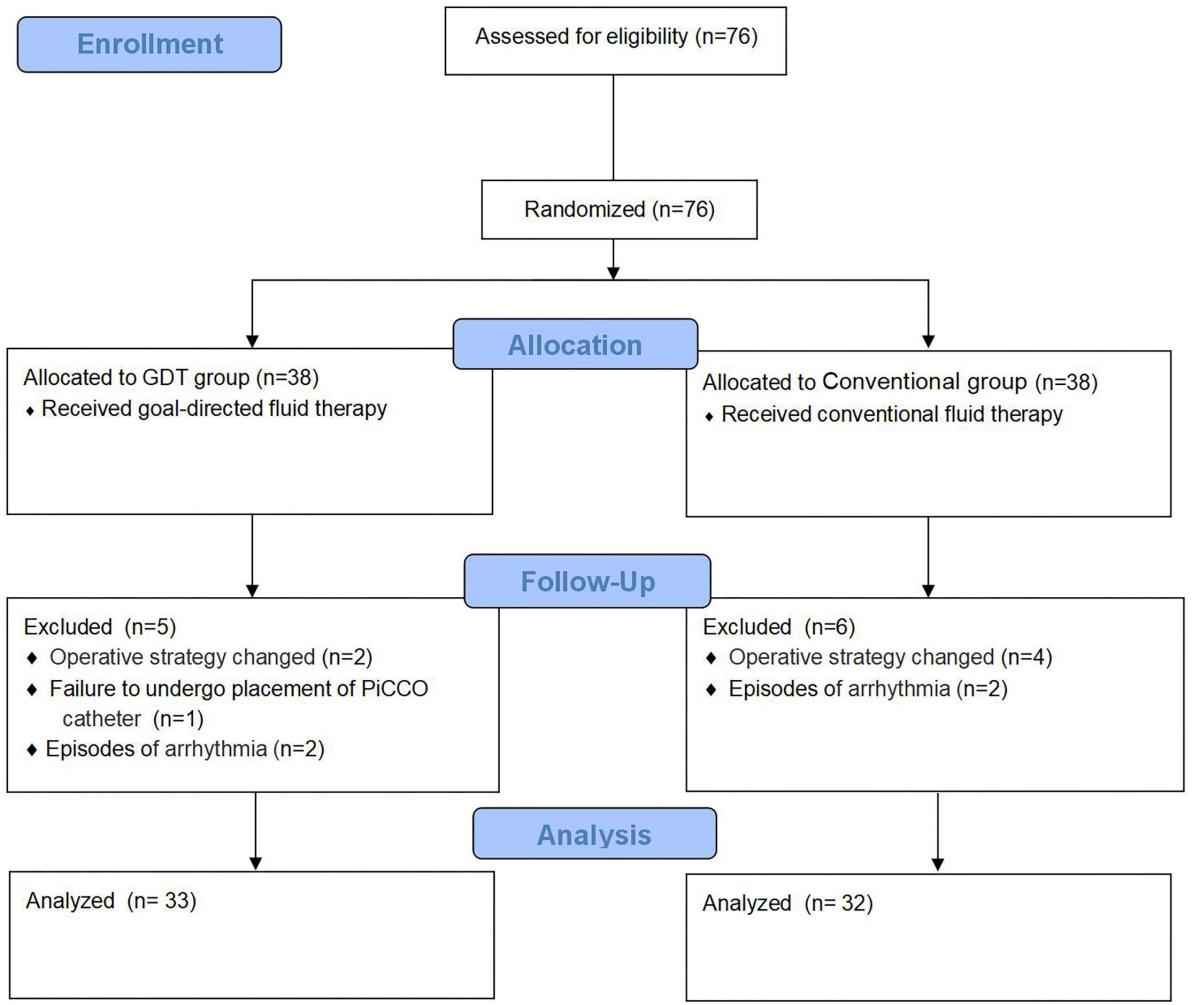
Flow chart.

From November 2017 to December 2018, 76 patients were screened in this study. Eleven patients were excluded because of changes in surgical approach (6 patients), new-onset intraoperative arrhythmia (2 patients with atrial fibrillation, and 2 patients with supraventricular tachycardia), and failure to cannulate the PiCCO catheter in 1 patient. Overall, 65 patients (*n* = 33 in the GDT group and *n* = 32 in the conventional group) were included in the analysis. A CONSORT diagram is shown in Figure 1. Baseline characteristics and surgical data were comparable between the two groups (Table 1).

**Table 1:** Demographics and clinical characteristics of the study population.

	**GDT**	**Conventional care**
	**(*n* = 33)**	**(*n* = 32)**
Age (years)	69 ± 3	70 ± 5
Sex (male/female)	28/5	23/9
BMI (kg/m^2^)	23.2 ± 2.9	22.6 ± 2.8
ASA classification (I/II/III/IV)	9/22/2/0	10/18/4/0
LVEF (%)	64 ± 1	64 ± 2
FVC (L)	2.9 ± 0.7	3.0 ± 0.6
FEV1 (L)	2.2 ± 0.6	2.4 ± 0.4
Smoking history (yes, n)	16	13
History of hypertension (yes, n)	15	14
History of diabetes mellitus (yes, n)	2	0
Preoperative neoadjuvant radiotherapy (yes, n)	5	3
Preoperative neoadjuvant chemotherapy (yes, n)	4	3
Preoperative hemoglobin (g/L)	139 ± 17	131 ± 19
Preoperative total plasma protein level (g/L)	71 ± 4	69 ± 5
Preoperative plasma albumin level (g/L)	41 ± 3	40 ± 3
**Surgical approach**		
Robot-assisted McKeown esophagectomy (n)	9	9
VATS-assisted esophagectomy (n)	24	23
Duration of surgery (min)	265 ± 46	258 ± 40
Duration of artificial pneumothorax (min)	87 ± 28	86 ± 24
Intraoperative minimum temperature (°C)	36.0 ± 0.4	36.2 ± 0.5
Preoperative fasting time (hours)	14 ± 3	14 ± 3
Tumor size (cm)	3 ± 1.6	4 ± 2.0
**Tumor stage**		
I/II/III/IVa	3/13/16/1	3/6/20/3
**Tumor location**		
Upper/middle/lower	1/14/18	2/19/11

*Data are presented as the means ± SDs or n. LVEF, left ventricular ejection fraction; FVC, forced vital capacity; FEV1, forced expiratory volume in 1 second; VATS, video-assisted thoracoscopic surgery. P <0.05 was considered statistically significant*.

### Postoperative Complications and Mortality

None of the patients died during postoperative hospitalization. In the primary analysis, the incidence of postoperative complications was not significantly different between groups (*P* = 0.92). 12 (36.4%) patients in the GDT group and 12 (37.5%) in the conventional group had at least one postoperative complication. The incidence of anastomotic leakage was 9.1% (3/33) in the GDT group and 21.8% (7/32) in the conventional group (*P* = 0.18). The incidence of postoperative pulmonary complications was 21.2% (7/33) in the GDT group and 21.8% (7/32) in the conventional group (*P* = 0.94). Furthermore, 3 cases (9.3%) of postoperative myocardial injury occurred in the GDT group and 6 cases (18.8%) occurred in the conventional group (*P* = 0.26). The incidence of hoarseness and chylothorax was not different between the two groups (Table 2).

**Table 2:** Postoperative complications and mortality.

	**GDT**	**Conventional care**	***RR* (Conventional vs. GDT)**	***P-*value**
	**(*n* = 33)**	**(*n* = 32)**	**(95% CI)**	
Postoperative complications (n, %)	12 (36.4%)	12 (37.5%)	1.03 [0.55–1.94]	0.92
Anastomotic leakage (n, %)	3 (9.1%)	7 (21.9%)	2.40 [0.68–8.50]	0.18
Pulmonary complications (n, %)	7 (21.2%)	7 (21.9%)	1.03 [0.40–2.60]	0.94
Hoarseness (n, %)	3 (9.1%)	3 (9.4%)	1.03 [0.22–4.74]	1.00
Chylothorax (n, %)	1 (3%)	3 (9.4%)	3.09 [0.34–28.21]	0.35
Postoperative myocardial injury	3 (9.3%)	6 (18.8%)	2.06 [0.56–7.55]	0.26
**Troponin level (μg/ml)**				
Preoperative The 1st day after surgery The 3rd day after surgery	0.01 ± 0.04 0.03 ± 0.03 0.01 ± 0.01	0.01 ± 0.01 0.09 ± 0.33 0.19 ± 0.90		0.59 0.30 0.28
Postoperative mortality	0	0		-
Postoperative length of stay (day)	13 ± 7	12 ± 7		0.52

*Data are presented as the means ± SDs or n (%). Postoperative myocardial injury was defined as troponin level > 0.06 μg/ml (99% UPL)*.

^*^T-test was used to compare continuous variables, and Chi-square test or Fisher's Exact test was used to compare categorical variables, as appropriate. Repeated measured ANOVA was used to compare troponin levels. For primary end points, P <0.05 was considered statistically significant.

### Secondary Outcomes

The total fluid volume did not differ between the two groups (2,192 ± 469 ml in GDT group vs. 2,201 ± 337 ml in the conventional group, *P* = 0.92). However, the ratio of crystalloid to colloid was significantly different (GDT vs. conventional, 1.3 [1.0, 2.3], 7.3 [6.0, 8.9], *P* <0.05). The intraoperative infusion volume of colloidal solution in the GDT group was higher than that in the conventional group (874 ± 369 vs. 270 ± 67 ml, *P* <0.05), and the volume of crystalloids was less than that in the conventional group (1,318 ± 386 vs. 1,937 ± 334 ml, *P* <0.05). There was no significant difference in operation time, duration of CO_2_ artificial pneumothorax, preoperative fasting time, intraoperative blood loss or intraoperative urine output between the groups (Table 3).

**Table 3:** Intraoperative fluid administration and surgical-related parameters.

	**GDT**	**Conventional care**	***P-*value**
	**(*n* = 33)**	**(*n* = 32)**	
Blood loss (ml)	230 ± 88	206 ± 25	0.14
Intraoperative urine output (ml)	442 ± 197	403 ± 239	0.46
Total intraoperative fluid volume (ml)	2,192 ± 469	2,201 ± 337	0.92
Crystal solution (ml)	1,318 ± 386	1,937 ± 334	0.01^*^
Colloidal solution (ml)	874 ± 369	270 ± 67	0.01^*^
Proportion of crystal to colloid	1.3 [1.0, 2.3]	7.3 [6.0, 8.9]	0.01^*^
Concentrated red cells (ml)	0 [0,0]	0 [0,0]	0.601
Norepinephrine (n)	17	23	0.09
**Urine (ml)**			
On the day of surgery	1,448 ± 811	1,370 ± 661	0.67
On the 1st day after surgery	1,678 ± 564	1,529 ± 415	0.23

*Data are presented as the means ± SDs, Median [IQR] or n*.

* *T test was used to compare continuous variables, Mann-Whitney U test was used to compare nonparametric outcomes and Chi-square test was used to compare categorical variables, as appropriate. Repeated measured ANOVA was used to compare urine output*.

The use of norepinephrine was not significantly different (51% vs. 72%, GDT vs. conventional group, *P* = 0.09), and nasopharyngeal temperature at the end of surgery was similar (36.0 ± 0.4 vs. 36.2 ± 0.5°C, GDT vs. conventional group, *P* = 0.05). Urine output on the day of surgery (1,448 ± 811 vs. 1,370 ± 661 ml, GDT group vs. conventional group = 0.67) and the 1st day after surgery (1,678 ± 564 vs. 1,529 ± 415 ml, GDT vs. conventional group, *P* = 0.23) did not differ between the two groups (Table 3).

Table 4 showed the hemodynamics and blood gas of the two groups. Thirty minutes after the establishment of artificial pneumothorax, the GDT group showed higher MAP (78 ± 13 vs. 72 ± 7 mm Hg, *P* <0.05) and central venous pressure (CVP: 17 ± 4 vs. 15 ± 4 mm Hg, *P* <0.05) and lower blood lactate levels (0.7 ± 0.2 vs. 1.0 ± 0.3 mmol/L, *P* <0.05) than the conventional group. Cerebral oxygen monitoring was not different between the two groups (*P* > 0.05).

**Table 4:** Hemodynamic, cerebral oxygen and blood gas parameters during operation.

	**Before**	**Initiation of**	**30 min after**	**30 min after**	**The end**
	**induction**	**pneumothorax**	**pneumothorax**	**the pneumoperitoneum**	**of surgery**
**MAP (mmHg)**				
GDT	103 ± 15	84 ± 20	78 ± 13	88 ± 11	85 ± 11
Conventional care	103 ± 14	83 ± 16	72+ 7	85 ± 14	86 ± 14
*P*-value	0.94	0.75	0.01	0.37	0.74
**CVP (mmHg)**				
GDT	/	7+ 3	17+ 4	14+ 5	8+ 3
Conventional care	/	7+ 3	15+ 4	12+ 5	7+ 3
*P*-value	/	0.43	0.01	0.11	0.08
**HR (beats/min)**				
GDT	76 ± 13	56+ 8	72 ± 11	68 ± 10	67 ± 10
Conventional care	72 ± 11	58+ 7	73 ± 11	67 ± 12	67 ± 10
*P*-value	0.19	0.31	0.75	0.80	0.91
**Cerebral oxygen (left/right) (%)**				
GDT	68 ± 5/67 ± 5	70 ± 4/69 ± 5	67 ± 6/65 ± 6	68 ± 6/66 ± 6	67 ± 6/64 ± 7
Conventional care	69 ± 6/67 ± 7	72 ± 5/69 ± 4	67 ± 5/64 ± 5	69 ± 5/66 ± 5	69 ± 6/66 ± 6
*P*-value	0.57/0.90	0.09/0.54	0.91/0.60	0.33/0.88	0.17/0.20
**Blood gas**				
**PH**				
GDT	/	7.41 ± 0.05	7.26 ± 0.05	7.30 ± 0.07	7.38 ± 0.05
Conventional care	/	7.41 ± 0.04	7.27 ± 0.05	7.33 ± 0.04	7.40 ± 0.03
*P*-value	/	0.95	0.18	0.11	0.12
**BE**				
GDT	/	−0.3 ± 1.6	−3.0 ± 1.9	−2.9 ± 1.8	−1.7 ± 1.4
Conventional care	/	−0.4 ± 1.3	−2.5 ± 1.5	−2.3 ± 1.0	−1.4 ± 1.5
*P*-value	/	0.77	0.22	0.06	0.34
**PaO**_**2**_ **(mmHg)**				
GDT	/	390 ± 91	202 ± 101	205 ± 70	193 ± 61
Conventional care	/	394 ± 69	212 ± 103	216 ± 56	224 ± 52
*P*-value	/	0.83	0.69	0.46	0.03
**PaCO**_**2**_ **(mmHg)**				
GDT	/	38 ± 6	57 ± 9	49 ± 8	40 ± 6
Conventional care	/	38 ± 5	55 ± 9.0	46 ± 7	38 ± 4
*P*-value	/	0.66	0.43	0.15	0.10
**Levels of blood glucose (mmol/L)**				
GDT	/	6.3 ± 0.9	7.3 ± 1.2	7.5 ± 1.3	7.6 ± 1.4
Conventional care	/	5.8 ± 0.9	6.9 ± 1.2	7.1 ± 1.2	7.1 ± 1.5
*P*-value	/	0.08	0.22	0.14	0.17
**Blood lactic acid level (mmol/L)**				
GDT	/	0.9 ± 0.2	0.7 ± 0.2	0.8 ± 0.2	0.8 ± 0.2
Conventional care	/	0.9 ± 0.3	1.0 ± 0.3	0.9 ± 0.3	0.8 ± 0.3
*P*-value	/	0.72	0.01	0.03	0.81
**Hematocrit (%)**				
GDT	/	41 ± 6	39 ± 6	37 ± 6	37 ± 6
Conventional care	/	39 ± 6	38 ± 5	37 ± 5	37 ± 5
*P*-value	/	0.21	0.85	0.96	0.88

*Data are presented as the means ± SDs*.

*Repeated measured ANOVA was used to compare continuous variables at different time points*.

## Discussion

In this randomized trial of elderly patients receiving minimally invasive esophagectomy, we found that SVV-GDT using PiCCO did not reduce the incidence of postoperative complications compared with conventional fluid treatment. The total fluid volume did not differ between the two groups, but the major difference between the groups was the higher volume of colloid with GDT treatment. There were no differences in intraoperative hemodynamics, cerebral oxygen and blood gas variables or postoperative length of stay.

The composite of postoperative complications occurred in nearly 40% of elderly patients, which is consistent with the result from Hikasa et al. (4). Among these complications, pulmonary complications, myocardial injury and anastomotic leakage constituted the most common complications after minimally invasive esophagectomy in this trial, in line with previous reports (16–18). None of patients died, and the average postoperative length of stay was ~12–13 days.

We found that the incidence of joint postoperative complications or each component of predefined complications was similar between the conventional fluid treatment and GDT groups. There is no consensus reported in the literature in terms of “goal-directed fluid therapy” (19). Goal-directed fluid strategies are usually recommended in esophageal cancer surgery, as they have been proven to reduce the incidence of perioperative complications and shorten the length of stay in abdominal surgery and orthopedic surgery (17–20). However, our results did not show a benefit of SVV-GDT in minimally invasive esophagectomy. Our results are supported by a recent trial (20) which found goal-directed therapy during esophageal resection didn't reduce the incidence of postoperative complications. Denise et al. also demonstrated that implementation of GDT during esophagectomy was not associated with reductions in overall morbidity, mortality or hospital length of stay (21).

Through our study, the total amounts of fluid administration were similar between the groups. Under the guidance of SVV, the average volume of intraoperative fluid in GDT was ~2,000 ml (nearly 7 ml/kg/h), comparable to that of conventional fluid treatment. Interestingly, we found that patients received over five times the colloids in GDT than in conventional treatment. In a retrospective analysis during esophageal cancer surgery, the higher crystal-to-colloid ratio was related to the increased risk of postoperative respiratory complications, AKI, infections and gastrointestinal complications (20). Inconsistently, different crystal-to-colloid ratios were reported to have no influence on postoperative complications in laparoscopic surgery (19). In our study, although the GDT group had a lower crystal-colloid ratio than the conventional group, the incidence of anastomotic leakage, postoperative pulmonary complications, postoperative myocardial injury and other complications did not differ. One possible explanation is that the strategy of SVV optimization based on PiCCO has inherent limitations, especially in thoracic surgery (21, 22). The usefulness of hemodynamic variables, such as SVV, is questioned in thoracoscopic surgery and one-lung ventilation when using small tidal volumes and CO_2_ artificial pneumothorax. Our data showed that the cardiac index decreased significantly from 2.0 to 1.6, while SVV increased from 12 to 21 when CO_2_ pneumothorax started. This change of SVV may lead to more frequent colloid administration, contributing to changes in the crystal-colloid ratio.

During the thoracic procedure, CVP increased dramatically after initiating artificial pneumothorax, and temporary hypercapnia occurred in both groups because of the CO_2_ pneumothorax and lower tidal volume ventilation. Notably, 30 min after the establishment of artificial pneumothorax, the GDT group showed higher MAP and lower blood lactate levels. However, in the abdominal phase, the acid-base status in both groups returned to normal, without the need to correct metabolic acidosis. There was no difference in the total dose of norepinephrine, intraoperative hemodynamics, lactate level or postoperative length of stay between the two groups.

There are several limitations in this study. First, the sample size may not have enough power to draw conclusions. We calculated our sample size based on an expected 75% reduction in postoperative complications, from 40 to 10%. However, the actual incidence in the present study was ~37% in both of the groups, which was close to our expectation. We assume it is unlikely that an increased sample size would have resulted in a favorable difference. Second, we only implemented the GTD strategy during surgery. Since fluid therapy is among the most challenging and important tasks that clinicians face on a daily basis, it is ideal to maintain intravascular euvolemia throughout the perioperative period. Fluid management preoperatively or after surgery may also affect the incidence of postoperative complications (21–23). Third, we did not use PiCCO to monitor patients in the control group; therefore, we do not know the effect of conventional fluid administration on cardiac output or other hemodynamic parameters when CO_2_ pneumothorax was initiated. Fourth, when using small tidal volumes and CO_2_ artificial pneumothorax in MIE, the accuracy of implementing SVV-GDT by PiCCO remains questioned (16, 24–26). Finally, although we set the correct target to follow during the intraoperative period, the quantity and type of fluids to administer remain under debate (27).

In summary, we found that colloid-based SVV optimization during GDT did not reduce the incidence of short-term postoperative complications in elderly patients receiving minimally invasive esophagectomy. Owing to the small sample size, the conclusion should be interpreted with caution.

## Data Availability Statement

The raw data supporting the conclusions of this article will be made available by the authors, without undue reservation.

## Ethics Statement

The studies involving human participants were reviewed and approved by Shanghai Chest Hospital Institutional Review Board, Shanghai, China. The patients/participants provided their written informed consent to participate in this study.

## Author Contributions

WT, YQ, MX, and JW contributed to conception and design of the study. WT organized the database and wrote the first draft of the manuscript. YQ performed the statistical analysis. YQ, HL, and JW wrote sections of the manuscript. All authors contributed to manuscript revision, read, and approved the submitted version.

## Funding

This work was supported by Shanghai Municipal Commission of Health (202040200) and Project 82071233 supported by the National Natural Science Foundation of China.

## Conflict of Interest

The authors declare that the research was conducted in the absence of any commercial or financial relationships that could be construed as a potential conflict of interest.

## Publisher's Note

All claims expressed in this article are solely those of the authors and do not necessarily represent those of their affiliated organizations, or those of the publisher, the editors and the reviewers. Any product that may be evaluated in this article, or claim that may be made by its manufacturer, is not guaranteed or endorsed by the publisher.
