# Supplementary material for: Stroke Volume Variation-Guided Goal-Directed Fluid Therapy Did Not Significantly Reduce the Incidence of Early Postoperative Complications in Elderly Patients Undergoing Minimally Invasive Esophagectomy: A Randomized Controlled Trial
Source: Front Surg. 2021 Dec 6;8:794272. doi: 10.3389/fsurg.2021.794272 (PMC8685214; doi:10.3389/fsurg.2021.794272)
Supplement: Supplementary file 1 [file Table_1.DOCX]

Supplemental table 1

| Melbourne Group Scale |
| --- |
| Temperature >38 ◦C |
| White cell count >11.2 or respiratory antibiotics |
| Physician diagnosis of pneumonia or chest infection |
| Chest X-ray report of atelectasis/consolidation |
| Production of purulent (yellow/green) sputum differing from preoperative |
| Positive signs on sputum microbiology |
| SpO2 <90% on room air |
| Re-admission to or prolonged stay (over 36 hours) on the intensive care unit/high dependency unit for respiratory problems |

PPC = four or more positive variables
