# Supplementary material for: Stroke Volume Variation-Guided Goal-Directed Fluid Therapy Did Not Significantly Reduce the Incidence of Early Postoperative Complications in Elderly Patients Undergoing Minimally Invasive Esophagectomy: A Randomized Controlled Trial
Source: Front Surg. 2021 Dec 6;8:794272. doi: 10.3389/fsurg.2021.794272 (PMC8685214; doi:10.3389/fsurg.2021.794272)
Supplement: Supplementary file 2 [file Table_2.docx]

**Supplemental table 2. Hemodynamic parameters measured by PiCCO in the GDT group at different time points**

|  | Before induction | Initiation of pneumothorax | 30 min after pneumothorax | 30 min after the pneumoperitoneum | The end of surgery |
| --- | --- | --- | --- | --- | --- |
| CI | 2.01±0.51 | 1.61±0.31 | 2.31±0.58 | 2.93±0.64 | 2.92±0.62 |
| SVV | 11.8±4.2 | 21.2±4.5 | 12.6±6.7 | 8.4±3.6 | 6.9±2.8 |
| SVI | 34.7±7.9 | 25.3±6.2 | 32.5±8.3 | 43.4±8.2 | 43.9±8.9 |
| SVRI | 3520±1178 | 3915±1072 | 2696±606 | 2464±661 | 2375±648 |
| PVPI | 1.67±0.46 | 1.76±0.45 | 1.86±0.45 | 1.39±0.24 | 1.47±0.30 |
| GEDI | 683±131 | 658±138 | 662±139 | 788±135 | 773±164 |
| GEF | 20.9±4.6 | 16.7±4.9 | 19.9±4.6 | 21.6±3.5 | 22.3±4.5 |
| EVLWI | 8.0±2.0 | 8.2±1.9 | 8.6±2.4 | 7.7±1.5 | 7.9±1.2 |

CI =cardiac index, SVV= stroke volume variation, SVI =stroke volume index, SVRI= systemic vascular resistance index PVRI= pulmonary vascular permeability index GEDI= global end-diastolic volume index, GEF=global ejection fraction, EVLWI= extravascular lung water index
